# Coercive compliance? Anti-doping systems in tennis and athlete mental health

**DOI:** 10.3389/fspor.2025.1636161

**Published:** 2025-07-16

**Authors:** Jill Colangelo, Alexander Smith, Malte Christian Claussen, Joao Mauricio Castaldelli-Maia, Michael Liebrenz

**Affiliations:** ^1^ Department of Forensic Psychiatry, University of Bern, Bern, Switzerland; ^2^ Research Group Sports Psychiatry, Center for Psychiatric Research, Department of Adult Psychiatry and Psychotherapy, Psychiatric University Clinic Zurich, University of Zurich, Zurich, Switzerland; ^3^ Clinic for Depression and Anxiety, Psychiatric Centre Muensingen, Muensingen, Switzerland; ^4^ Department of Psychiatry, Medical School, University of São Paolo, Sao Paolo, Brazil

**Keywords:** tennis, mental health, sports psychiatry, anti-doping, law suits

## Abstract

Recently, a group of professional tennis players (i.e., the Professional Tennis Players Association) filed a legal case against several governing bodies in the sport. This suit intends to challenge the alleged disregard for athlete wellbeing when enforcing anti-doping policy, as this can engender adverse effects even in unintentional or unproven cases. This complaint is set against the background of several high-profile doping proceedings in tennis, which have further revealed potential inconsistencies in integrity investigations and processes. Accordingly, the purpose of this article is to explore the possibly harmful conditions for athletes described in this litigation, as well as to acknowledge the need for multi-faceted support among professional players. In doing so, this perspective paper also draws attention to the need for fairness in professional sport, alongside proposing ways in which sport psychiatrists and sports medicine physicians can advise and advocate for education for players, other healthcare specialists, and governing bodies.

## 1 Introduction

In January 2025, twelve elite-level tennis players, collectively supported by the Professional Tennis Players Association (PTPA), initiated a civil class-action antitrust lawsuit targeting four dominant bodies in tennis (i.e., Case 1:25-cv-02207, United States District Court, Southern District of New York) (1). Namely, the listed defendants were the Association of Tennis Professionals (ATP), the Women's Tennis Association (WTA), the International Tennis Federation (ITF), which are the foremost governing entities in the sport (1). The International Tennis Integrity Agency (ITIA), which is responsible for overseeing anti-doping according to the World Anti Doping Agency (WADA) regulations and anti-corruption in tennis, was also named as a defendant (1). This litigation was instigated following numerous complaints from current professional players, which they argued did not result in productive dialogues, together with a perceived disregard for their overall wellbeing (2).

The plaintiffs contend that the ATP, WTA, ITF, and ITIA operate as a “cartel”, enforcing overly rigid and interlocking constraints that exploit players, compromise physical and mental health, and violate personal rights (1, 2). Amongst the wide-ranging claims of suppressed earnings and monopolistic controls, the suit cites apparently invasive disciplinary practices in anti-doping investigations as examples of abuse (3). Notably, these allegations carry particular relevance for sports psychiatry and sports medicine, given the well-documented vulnerabilities of elite athletes as well as the recognition that regulatory systems may exacerbate psychological stressors under certain conditions (4–7).

At the time of writing, it should be acknowledged that the defendants vehemently repudiate these assertions and the case remains ongoing. Nevertheless, in May 2025, the Court ruled that these organisations could not retaliate against athletes who choose to participate in the suit, demonstrating the potentially precarious relationship between players and the governing bodies (1, 8).

Amidst this context, this article analyses this situation from the perspective of sports psychiatry. Specifically, it explores how anti-doping enforcement can intersect with mental health in elite-level sports and how such issues may introduce complexities for both medical ethics and the responsibilities of healthcare specialists interacting with athletes.

## 2 Anti-doping in tennis and the PTPA complaint

As in other sports, there are prevailing negative attitudes toward doping in tennis, but the use rates of banned substances are believed to be comparatively low (9, 10). That said, there is scarce research on the consumption trends of performance enhancing drugs in tennis, though the most aggressive usage reportedly occurs in lower national rankings and can dramatically affect careers (9). Additionally, the accuracy of the information received by the athlete regarding the assumption of substances and their perceived effects has been debated (10). Relatedly, questions have been raised over the effectiveness of drug testing to reveal illegal usage and whether or not this functions as a legitimate deterrent (10).

Despite this, the PTPA argues in their new case “on behalf of the entire player population” that ITIA procedures contravene due process rights for tennis players and engender coercive and psychologically harmful conditions (1). The plaintiffs accuse the ITIA of conducting arbitrary and invasive investigations, such as numerous drug tests and compulsory disclosure of personal data (e.g., phone searches) (1, 2). Following the announcement of this case, other regulatory clarifications surfaced, including an ITIA policy requiring players selected for drug testing to shower under direct observation if they choose to wash before providing a sample (11). Though the ITIA indicated that showering “is not an entitlement”, this prompted a backlash from certain fans and media outlets for perceived overreach and impinging on players' rights (11).

Moreover, the ITIA is accused by the PTPA of suspending players without due process based on “flimsy or fabricated” evidence (2). According to the plaintiffs, these actions generally occur without robust appeal pathways or adequate independent oversight (1). Per the PTPA, athletes have been required to sign wide-ranging waivers and arbitration agreements, supplanting standard legal protections and heightening risks for investigatory overreach (1). For the PTPA, the current situation invokes concomitant concerns about “monopolistic control” within tennis (1); in this regard, they contend that players' ability to compete professionally is inherently associated with rankings and tournament eligibility, which are contingent on compliance with overarching regulations (1, 2). Resultantly, the refusal to cooperate with ITIA procedures may jeopardise players' careers and reputations (2).

This case comes off the back of recent high-profile doping allegations against the men's World Number One, Jannik Sinner, and the way in which these official proceedings arguably may have contrasted with other athletes' experiences (12, 13). Earlier high-profile doping cases have transpired in tennis centred around other substances, and these also implicated successful players; in more recent incidents, like the one involving Sinner, athletes have explicitly acknowledged encountering psychological difficulties (10, 14). Others have cited his preferential treatment due to potential financial implications and access to superior legal representation (14). Notably, these conditions were referenced in support of the PTPA legal action (15). Hence, the PTPA's calls for fairness, accountability, and structural change has implications both for the sport of tennis and the wellbeing of its athletes.

## 3 Doping and athlete mental health in elite-level sports

Despite the fact that the sport of tennis has been known to confer physical and mental health benefits, the lawsuit describes a culture in elite-level domains that may adversely affect individual wellbeing (16). Indeed, irrespective of the perceived and alleged unfairness in anti-doping protocols, elite-level tennis players have suggested that the substantial pressure and scrutiny in anti-doping cases alone may be damaging to psychological status and performance (17). By corollary, the litigants indicate that anti-doping and integrity investigations can cause psychological harms (1, 2). This is pertinent since scientific literature shows that elite athletes are already be prone to elevated risk factors for various psychiatric disorders (4). Similar patterns have been identified in professional tennis, which comprises distinctive stressors, like competitive pressures, fatigue, exposure to abuse, and busy travel schedules (18).

Notably, Nick Kyrgios, one of the players named in the PTPA action, has highlighted his mental health challenges, conceding: “My life was kind of spiraling out of control, drinking every single night” (19). These concerns are not unknown to international federations and integrity bodies; WADA, which is responsible for establishing rules for drug-free sport, has published documentation reviewing the correlation between doping and psychiatric disorders (20). Accordingly, sport-specific psychiatric vulnerabilities in tennis could conceivably be exacerbated by lengthy regulatory processes involving suspected doping and possible career sanctions (5–7). In this regard, the former world number one in women's tennis, Simona Halep, described experiencing insomnia and anxiety as she contested a doping charge that was eventually deemed to be unintentional (7).

Additionally, athletes beyond tennis have been subjected to harassment and online abuse amidst doping claims (21, 22). Other tennis players have expressed intense fear of inadvertently consuming banned substances, together with not having the financial means to combat allegations should they test positive (23); again, this contrasts with the reports of certain athletes' ability to obtain high-cost legal representation contingent on their wealth and success.

Suspensions due to doping can be lengthy, even if an athlete is not found to have intentionally used a performance-enhancing drug (24, 25). Whilst these measures can help to protect fair play, in elite level tennis, they have caused players to experience anxiety and depression, even when the substances unintentionally used would not result in improved performance (25, 26). In other sporting frameworks, suspensions have reportedly entailed severe mental health consequences, although again enforced with the aim of promoting fairer competition (27, 28). Thus, some have accentuated a duty of care and the need for supportive measures for athletes who have received a sanction to enable their rehabilitation or to more successfully handle the transition away from competition (29).

Further, certain d that may be necessary for athletes for physical and/or mental health purposes outside of sports cannot be utilised without a therapeutic use exemption (30). Female infertility treatments, medications to mitigate sleep disorders, and drugs that manage inflammatory bowel disease are among those that would otherwise be forbidden without a Therapeutic Use Exemption (TUE) from WADA (30). From a psychiatric perspective, stimulants used to treat ADHD are also prohibited without a TUE. Treating an athlete-patient with a pharmaceutical that requires a TUE warrants consideration about prudent use in competitive contexts, as well as whether it is ethical to withhold appropriate treatment in order to comply with WADA regulations (5). These decisions may be compounded by issues like drug contamination and side effects due to athletes' distinctive physiology, while maintaining individual dignity and wellbeing (31, 32).

As may stimulants often improve focus and concentration, issues have been raised in tennis about the number of elite-level players taking them illegitimately and the increase in TUE requests (33, 34). These questions have been invoked as other commentators have portrayed doping in sports as an “epidemic” (35). Nonetheless, it is concerning that the legitimate use of stimulants could be subject to stigma, as some athletes have been vocal about their ADHD diagnoses and the benefits of medication for their overall wellbeing (36). Likewise, in other sports, athlete TUEs for ADHD psychopharmaceuticals were estimated to be comparatively lower than medication rates in the general population (6).

## 4 The role of sports psychiatrists and sports medicine physicians in anti-doping

Whilst integrity systems are essential for ensuring fairness and public trust in sport, they must be balanced against the mental health needs and human rights of athletes, as the ITIA complaint implies. Claims of a lack of fair play undermines the concepts of sportsmanship and erodes motivation to participate in and follow sport more broadly (37). That said, the allegations about ITIA in the PTA lawsuit draw attention to risks for an over-corrective disciplinary architecture, which the plaintiffs argue prioritises institutional reputation and interest instead of individual welfare (1, 2). Therefore the absence of procedural standardisation or legally mandated psychological support during these cases represents a significant oversight, especially given what is known about the psychosocial, financial, and somatic toll of legal interactions (18, 29).

Beyond tennis, similar issues have been identified across different sports, where anti-doping systems operate with varying degrees of independence (or not at all) (5, 8, 38, 39). WADA has recognised these concerns, commissioning a review into the compatibility of anti-doping rubric with supranational human rights laws and how this relates to athletes (40). Equally, researchers have argued that modern anti-doping systems disproportionately emphasise repression and may entail complexities for physicians in adhering to the ethical principles of non-maleficence and patient confidentiality (8, 9). In particular, sports psychiatrists and sports medicine physicians needing to treat conditions with medications that are banned may be met with additional challenges as they seek to balance adequate care with anti-doping protocols (41).

To address these problems, alternative models have been proposed centred around harm reduction and prevention, which go beyond the scope of this paper (5, 8). Elsewhere, although not devoid of criticism, independent agencies have been instructed to handle anti-doping procedures on behalf of international federations to mitigate conflicts of interest. This includes the International Testing Agency (ITA), who have been delegated responsibilities for anti-doping by a growing number of sporting entities, notably the Union Cycliste Internationale (42). Such separation may serve as a partial framework for how procedural fairness can be promoted; nevertheless, to uphold athlete mental health, these structures must be supplemented by rigorous safeguards and dedicated psychiatric care (5, 29).

As the lawsuit against the ITIA and other tennis governing bodies continues, sports psychiatrists should proactively engage with the intersections of doping and athlete mental health since they are capable of navigating between law and medicine, and between ethics and enforcement (43). To that end, training schemes for specialists about the TUE process and legal obligations and compulsory mental health services for athletes in anti-doping proceedings can be promoted.

WADA's educational course for sports medicine physicians could be incorporated into curricula supported by the International Society for Sports Psychiatry to maintain adherence to protocols as well as continuity of care (43, 44). Broader dialogues are also necessary to ensure that the pursuit of integrity in sport does not come at the expense of athlete wellbeing. To avoid any additional pressures, standardized and transparent testing that aligns with human rights in every circumstance and the process for judgements on violations should be available to all those athletes investigated.

Research has shown both that education can reduce the likelihood that an athlete would seek to use a prohibited substance and that athletes have a right to that information (20, 45–47). As the WADA report on social psychology and doping suggests, earlier interventions and psychological counselling may be critical to decreasing athlete susceptibility (20). For example, training for athletes and additional mental health monitoring and care are areas of opportunity and growth for the Association of Tennis Professionals (ATP), as current support services may be inadequate (48). Likewise, the Women's Tennis Association can also build upon its existing programmes to facilitate access to education and assistance around the consumption of banned substances (49). Moreover, based on the International Olympic Committee's framework as set forth in its consensus statement on mental health, comprehensive recommendations and potentially useful procedures have been implemented by governing federations in other sports, which could provide a foundation for future procedural amendments (50, 51). Sports psychiatrists and sports medicine physicians can contribute to these projects to ensure their compliance with up-to-date evidence.

Finally, as awareness of the links between doping and compromised mental health becomes clearer, support structures should be implemented for vulnerable athletes in collaboration with sports psychiatrists and sports medicine physicians (52). Acknowledgment of the potential ethical dilemmas in justly carrying out testing is critical to the success of these initiatives (53). With this, difficulties will likely arise in attracting financial means for rehabilitative efforts. However, as the possibility of monetary gain has also impacted athlete susceptibility to using prohibited substances, influential stakeholders could be encouraged to contribute to athlete wellbeing as a positive reflection on the sport of tennis more generally (47).

## 5 Conclusion

Public perception in professional sports is paramount to economic growth, and the subsequent financial impact of doping allegations can be sizeable (53, 54). It is therefore logical that governing bodies in tennis would seek to legitimize and protect the integrity of the sport. However, despite their fame and celebrity, elite-level athletes are still human beings with personal rights to privacy, equity, and wellbeing. Preserving their rights through standardized and transparent protocol should be prioritized, even in the face of ethical challenges.

To that end, sports psychiatrists and sports medicine physicians should contribute to ongoing dialogues about balancing sporting integrity with athlete wellbeing, as has been outlined above. An emphasis on dedicated education schemes, transparency about TUEs, better support systems for athletes, and advocacy for fairness in regulatory investigations represent just some of the initiatives that could be pursued to mitigate practical and professionals issues. Ultimately, we believe that the importance of and promoting the principles of fair play and clean sport should be balanced against supporting the improvement and sustainability of anti-doping protocols in elite-level competitions.

## Data Availability

The original contributions presented in the study are included in the article/Supplementary Material, further inquiries can be directed to the corresponding author/s.
